# *MsFtsH8* Enhances the Tolerance of PEG-Simulated Drought Stress by Boosting Antioxidant Capacity in *Medicago sativa* L.

**DOI:** 10.3390/plants13213025

**Published:** 2024-10-29

**Authors:** Ruyue Li, Xiangcui Zeng, Xueqian Jiang, Ruicai Long, Fei He, Xue Wang, Lin Chen, Qianwen Yu, Junmei Kang, Qingchuan Yang, Tianhui Yang, Zhongkuan Liu, Mingna Li

**Affiliations:** 1 Institute of Animal Sciences, Chinese Academy of Agricultural Sciences, Beijing 100193, China; lry15838083402@163.com (R.L.); xiangcui0212@163.com (X.Z.); dragongodsgod@163.com (R.L.); hefei0609@126.com (F.H.); wangxue01@caas.cn (X.W.); chenlin@caas.cn (L.C.); 0791@163.com (Q.Y.); kangjmei@126.com (J.K.); qchyang66@163.com (Q.Y.); 2 Institute of Grassland Research, Chinese Academy of Agricultural Sciences, Hohhot 010010, China; 3 Institute of Animal Sciences, Ningxia Academy of Agriculture and Forestry Sciences, Yinchuan 750002, China; 4 Institute of Agricultural Resources and Environment Research, Hebei Academy of Agriculture and Forestry Sciences, Shijiazhuang 050051, China; zhongkuanjh@163.com

**Keywords:** *Medicago sativa* L., FtsH, drought tolerance, ROS level, antioxidant

## Abstract

Drought is a major abiotic stress that limits the growth and yield of alfalfa, a vital forage legume. The plant metalloproteinase Filamentation temperature-sensitive H (FtsH) is an ATP- and Zn^2+^-dependent enzyme that plays a significant character in the plant’s response to environmental stress. However, its functional role in drought resistance remains largely unexplored. This study investigates the drought tolerance role of alfalfa *MsFtsH8* by analyzing the growth, physiology, and gene expression of overexpressing plants under drought conditions. The results demonstrated that both *MsFtsH8*-overexpressing *Arabidopsis* and alfalfa plants exhibited superior growth condition and enhanced membrane stability. The overexpressing alfalfa plants also showed reduced MDA levels, higher proline content, lower H_2_O_2_ accumulation, an increased activity of antioxidant-related enzymes (SOD, POD, and CAT) activity, and an elevated expression of antioxidant-related genes. These results indicated that the overexpression of *MsFtsH8* enhanced growth, improved osmotic regulation, reduced ROS levels, and increased antioxidative capacity, ultimately leading to greater drought tolerance in alfalfa. Our findings suggest that *MsFtsH8* mitigates oxidative damage caused by drought by modulating the plant’s antioxidant system, thus improving drought tolerance in alfalfa. This study provides a molecular basis and candidate genes for enhancing drought resistance in alfalfa through genetic engineering.

## 1. Introduction

Alfalfa (*Medicago sativa* L.) is the most important perennial legume forage globally, renowned for its high yield, protein content, and palatability, earning it the title “Queen of Forage” [1,2]. Alfalfa’s robust environmental adaptability and high yield, make it essential for improving soil fertility and regional ecosystems [2,3]. With an estimated 35 million hectares across 80 countries, alfalfa is a crucial resource for hay, silage processing, and grazing [4,5]. However, global warming and extreme weather conditions have intensified abiotic stresses, particularly drought, which leads to the accumulation of reactive oxygen species (ROS), causing oxidative damage to cells and impairing alfalfa’s growth [6]. Recent advances in molecular biology have facilitated the use of biotechnological tools, such as transgenics and gene editing, to improve drought tolerance in alfalfa. Alfalfa is moderately drought-tolerant due to its deep root system, yet the increasing frequency of drought events underscores the need for further genetic improvements to enhance this trait [6].

Proteases like FtsH, which are ATP- and Zn^2+^-dependent metalloproteinases, play a crucial role in maintaining protein homeostasis by degrading damaged proteins under stress conditions [7]. Studies have shown that *FtsH* genes are involved in various stress responses, including heat, drought, and cold. For example, *FtsH6* is induced in *Arabidopsis* under high-temperature stress [8], and *FtsH11* loss-of-function *Arabidopsis* is more sensitive to heat stress [9]. Under drought and high-temperature stress, the expression levels of *Arabidopsis FtsH2* and *FtsH8* were significantly elevated [10]. Under high light and high-temperature stress, *Arabidopsis FtsH1*, *FtsH5*, *FtsH6*, and *FtsH8* were significantly upregulated [11].

Furthermore, the lack of *AtFtsH11* resulted in heat sensitivity [9], and the deficiency of *ZmNEEDLE1*, a homolog of *AtFtsH10*, contributed to impaired growth and development under high-temperature conditions [12]. The knockdown of *CsFtsH5* resulted in hypersensitivity to cold stress [13], while the overexpression of *TsFtsH8* improved cold stress tolerance [14], and *AtFtsH4* could promote tolerance to long-term moderate heat stress [15]. Under abiotic stress conditions, FtsH degrades and removes the binding protein D1 damage in the PSII of plants, reduces the oxidative damage to the photosynthetic system proteins, maintains the stability and vitality of the PSII system, and helps plants adapt to and resist adverse stress conditions [7,8].

The expression abundance of FtsH8 protein increased significantly in alfalfa after salt stress, but did not change significantly in the relatively salt-sensitive *Medicago truncatula*, suggesting a role in stabilizing photosynthetic systems under stress [16]. Previous studies have demonstrated that the overexpression of *MsFtsH8* enhances salt tolerance by stabilizing photosynthetic proteins and boosting enzymatic activity [1]. However, its role in drought resistance remains unclear.

This study utilizes *MsFtsH8*-overexpressing *Arabidopsis* and alfalfa plants to explore the gene’s role in drought tolerance. By examining growth, physiological, and biochemical responses under drought stress, this study illustrates the function and mechanisms by which *MsFtsH8* confers drought tolerance. Our results provide a crucial candidate gene and lay a theoretical foundation for future genetic improvements in alfalfa.

## 2. Materials and Methods

### 2.1. Plant Materials and Growth Conditions

The *Medicago sativa* L. *cv* ‘Zhongmu-1’ and *Arabidopsis thaliana* Col-0 plant materials used in this study were supplied by our research group. The plants were grown in an intelligent artificial climate incubator under controlled growth conditions: 26 °C for 16 h of light/22 °C for 8 h of dark, 60% relative humidity, and a light intensity of 1200 μmol m^−2^s^−1^. Transgenic *Arabidopsis* and alfalfa plants overexpressing the *MsFtsH8* gene were generated in previous experiments [1,17]. The *MsFtsH8*-overexpressing line OE10 was selected based on its superior phenotype and physiological performance under salt stress [1,18].

### 2.2. Drought Stress Treatment and Sampling

In this study, to maintain uniform and consistent treatment conditions, mannitol and PEG were utilized to simulate drought stress.

For medium-grown *Arabidopsis*, seeds were sterilized with 10% sodium hypochlorite for 8 min, rinsed six times with ddH_2_O, and vernalized at 4 °C in darkness for two days in 300 μL ddH_2_O. The seeds were then sown on 1/2 Murashige and Skoog (MS) medium, 1/2 MS medium with 125 mM mannitol, and 1/2 MS medium with 150 mmol/L mannitol, and cultured vertically in growth Petri dishes (13 cm long × 13 cm wide). Each treatment had three biological replicates. After 15 days, growth was recorded, and root length and fresh weight were measured.

For soil-grown *Arabidopsis*, 10-day-old seedlings were transplanted into pots (8.5 cm long × 8.5 cm wide) filled with a 2:1 mixture of nutrient soil–vermiculite, with 3–4 seedling per pot, and grown for 30 days. Drought stress was applied by applying 40 mL of 500 mM mannitol solution per pot. Phenotypic changes were observed after three days, and growth, fresh weight, and relative conductivity were measured. Non-stressed plants watered with regular water served as controls, with three biological replicates per treatment.

For alfalfa, wild type (WT) and overexpressing line (OE10) plants were vegetatively propagated from cuttings. After 50 days, when roots were approximately 8 cm long, plants were transplanted to the nutrient solution (1/2 Hoagland: a nutrient solution formula containing nitrate, ammonium nitrate, magnesium sulfate, potassium sulfate, ferrous sulfate, boric acid, etc.) for hydroponic culture for 20 days, with the solution replaced every five days. Plants of uniform growth were then subjected to drought stress by transferring them to pots (27 cm × 21 cm × 7 cm) containing 1/2 Hoagland nutrient solution (control) and 1/2 Hoagland nutrient solution with 10% PEG6000 (treatment group). After three days, leaf dehydration and wilting were evident, and leaf samples were collected, frozen in liquid nitrogen, and stored at −80 °C for further analysis.

### 2.3. Physiological and Biochemical Measurements

For relative conductivity assays, 0.2 g fresh *Arabidopsis* leaf samples were washed with ddH_2_O and incubated in 10 mL of ddH_2_O at room temperature for five hours. The electrical conductivity of the solution was assayed as RC0 using a conductometer. After boiling at 100 °C for 20 min and cooling, the electrical conductivity was determined as RC15. The relative conductivity was calculated as RC0/ RC15. The other physiological and biochemical indicators related to stress resistance in alfalfa leaves were measured using plant stress resistance detection kits (Nanjing Jiancheng Technology Co., Ltd., Nanjing, China). The parameters assessed included malondialdehyde content (Cat# A003-1-1), proline content (Cat# A107-1-1), reactive oxygen levels, encompassing hydrogen peroxide content (Cat# A064-1-1), and anti-superoxide anion free radical activity (Cat# A052-1-1)), as well as antioxidant enzymes like catalase content (Cat# A007-1-1), superoxide dismutase content (Cat#A001-1-1), and peroxidase content (Cat# A08-3-1). Experiments were conducted referring to the instructions, with three biological replicates per treatment.

### 2.4. DAB and NBT Staining

Drought-stressed alfalfa leaves were rinsed with ddH_2_O and stained overnight at room temperature in the dark with 3,3-Diaminobenzidine tetrahydrochloride (DAB, 0.05 g DAB powder dissolved in 45 mL distilled water, 500 μL 1 M sodium dihydrogen phosphate, pH 3.8) solution and Nitrotetrazolium blue chloride (NBT, 0.1 g NBT powder dissolved in 50 mL 50 mM phosphate buffer) solution. The leaves were then boiled in anhydrous ethanol for 30 min in a water bath with gentle shaking, cooled to room temperature, and transferred to 60% glycerol on filter paper for imaging.

### 2.5. RNA Extraction, cDNA Synthesis, and qRT-PCR Analysis

Total RNA was extracted from alfalfa leaf samples using an Eastep Super total RNA extraction kit (Shanghai Promega Bioproducts Co., Ltd., Shanghai, China), with RNA concentration and integrity assessed by a micro-spectrophotometer (Implen NP80 Touch, Munich, Germany) and 1% agarose gel electrophoresis. RNA samples were adjusted to 200 ng/μL for cDNA synthesis using a UnionScript First-strand cDNA Synthesis Mix for qPCR (Beijing Jinsha Biotechnology Co., Ltd., Beijing, China). Gene expression analysis of *MsFtsH8*, catalase (CAT), and superoxide dismutase (SOD) was conducted using a Taq Pro Universal SYBR qPCR Master Mix (Nanjing Novozyme Biotechnology Co., Ltd., Nanjing, China) on a fluorescent quantitative PCR instrument (ABI 7300, Foster City, CA, USA). *MsACTIN2* (MSG0380016789.01) was used as the internal reference gene. Primers were designed online accessed on 1 April 2024 (https://www.ncbi.nlm.nih.gov/). *MsActin*-F:: 5′-CAAAAGATGGCAGATGCTGAGGAT-3′; *MsActin*-R: 5′-CATGCACCAGTATGACGAGGTCG-3′; (2) *MsSOD*-F: 5′-TGCCAAAGCTGATTCCTCAA-3′; *MsSOD*-R: 5′-TCACGAACAGGAGCCAGATT-3′; (3) *MsCAT*-F: 5′-AACAAGGCTGGGAAAGCAGT-3′; *MsCAT*-R: 5′-CGTGAGCAGGATAGGTCTTGA-3′. The amplification and melting curves were used to assess product specificity and efficiency. Transcript expression level was calculated using the 2^−ΔΔCt^ formula, with three independent biological replicates.

### 2.6. Statistical

Data were analyzed and visualized using Excel 2019 and GraphPad Prism 10.1.2. Statistical significance was tested by ANOVA (*p* ≤ 0.05), and results were shown as mean ± SEM (n = 3 for all groups).

## 3. Results

### 3.1. Drought Tolerance Analysis in MsFtsH8-Overexpressing Arabidopsis

**Figure 1:**
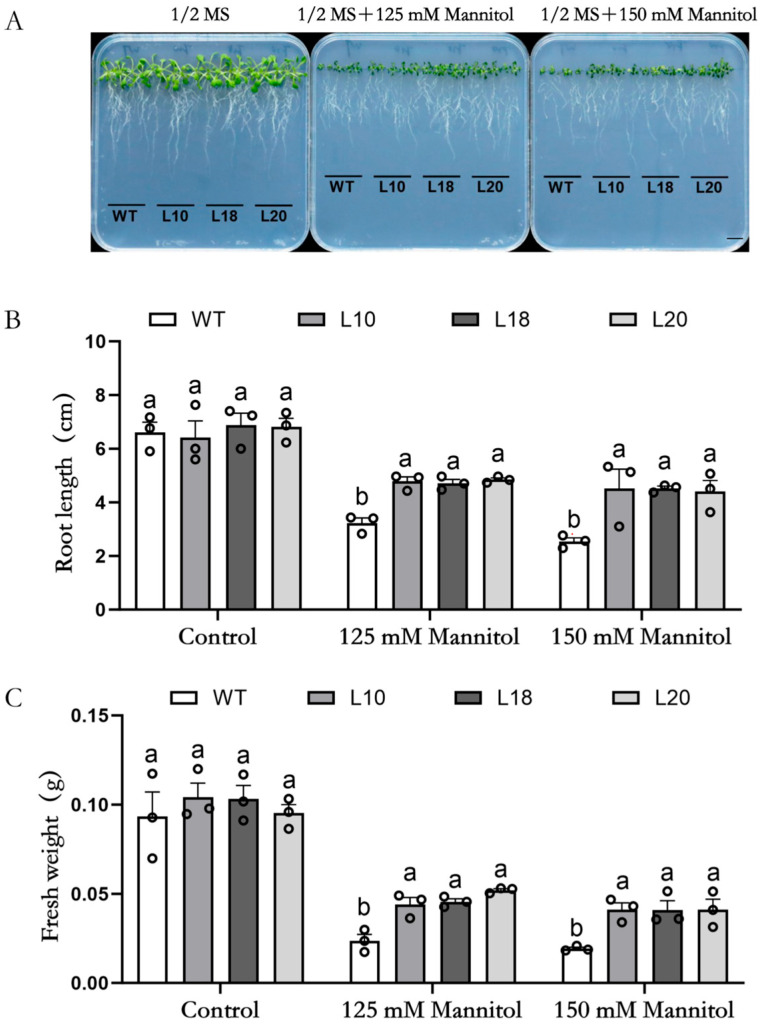
Growth phenotypes of *Arabidopsis thaliana* plants overexpressing the *MsFtsH8* gene under mannitol treatment. (**A**) Growth phenotype of *Arabidopsis* plants overexpressing *MsFtsH8* gene under mannitol stress. (**B**) Root length statistics of *Arabidopsis* plants overexpressing *MsFtsH8* under mannitol stress. (**C**) Fresh weight statistics of *Arabidopsis* overexpressing *MsFtsH8* under mannitol stress. WT *Arabidopsis* plants and *MsFtsH8*-overexpressing *Arabidopsis* plants L10, L18, and L20 were exposed to 1/2MS, 1/2MS + 125 mM Mannitol, and 1/2MS + 150 mM mannitol for 15 days. The bar value in the lower right corner of (**A**) is 1 cm. The circles in this figure represent the value of each replicate; n = 3 for all groups. The bars represent the SE. Bars marked with different lowercase letters denote statistically significant differences at *p* < 0.05 as assayed by ANOVA.

To assess the impact of the *MsFtsH8* gene on *Arabidopsis* growth under drought conditions, we compared WT *Arabidopsis* with overexpressing lines (L10, L18, and L20) grown in 1/2 MS medium supplemented with mannitol. Our results revealed that while WT and transgenic lines showed no significant difference in the control group, disparities emerged under mannitol stress (Figure 1A). Specifically, the mean root length in WT and overexpressing lines under control conditions was approximately 6.67 cm. However, under 125 mM mannitol, the root length of the overexpressing lines was around 4.78 cm, significantly greater than the 3.22 ± 0.20 cm observed in WT. Similarly, under 150 mM mannitol, the overexpressing lines maintained a mean root length around 4.48 cm, compared to 2.54 ± 0.14 cm in WT (Figure 1B). Fresh weight measurements showed that under control conditions, both WT and overexpressing lines had a mean fresh weight around 0.099 g. Yet, under 125 mM mannitol, overexpressing lines retained a fresh weight around 0.047 g, higher than WT by 0.024 ± 0.004 g. Under 150 mM mannitol, overexpressing lines maintained a fresh weight around 0.041 g, exceeding WT by 0.019 ± 0.001 g (Figure 1C). These findings suggest that *MsFtsH8* overexpression enhances drought tolerance in *Arabidopsis* by increasing root length and fresh weight under mannitol-induced stress.

**Figure 2:**
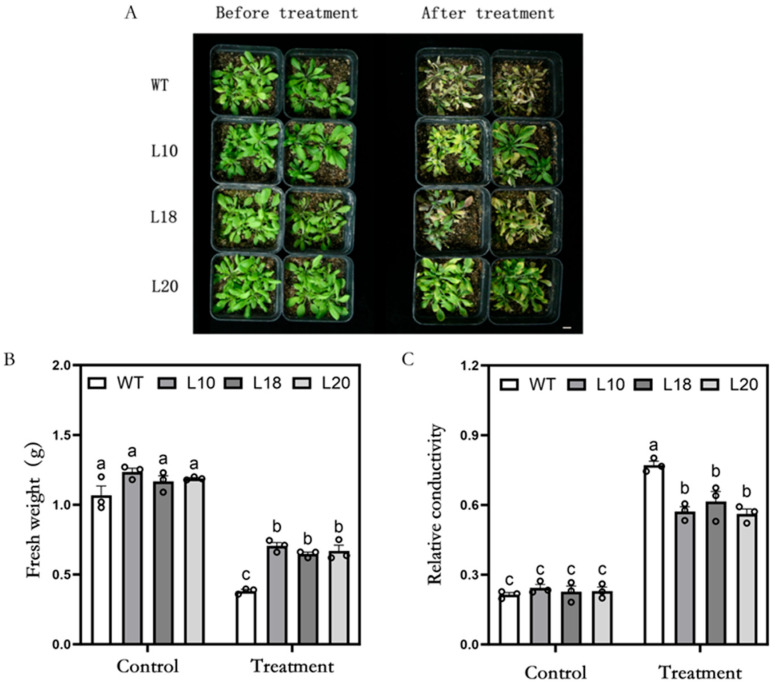
Growth phenotypes of *Arabidopsis thaliana* plants overexpressing *MsFtsH8* under mannitol treatment in soil culture. (**A**) Growth phenotype of *Arabidopsis thaliana* overexpressing *MsFtsH8* under mannitol stress in soil culture. (**B**) Fresh weight of *Arabidopsis* plants overexpressing *MsFtsH8* in soil culture under mannitol stress. (**C**) Statistics of relative conductivity of *Arabidopsis* plants overexpressing *MsFtsH8* under mannitol stress in soil culture. Forty-day-old plants with consistent growth were chosen and planted in pots, and each pot was watered with 40 mL of 500 mM mannitol solution for three days for treatment. The bar value in the lower right corner of (**A**) is 1 cm. The circles in this figure represent the value of each replicate; n = 3 for all groups. Bars marked with different lowercase letters denote statistically significant differences at *p* < 0.05 as assayed by ANOVA.

To further investigate the drought tolerance of *MsFtsH8*-overexpressing *Arabidopsis*, we subjected soil-grown WT and transgenic lines (L10, L18, and L20) to mannitol treatment. Both WT and transgenic lines exhibited similar growth under control conditions. However, under mannitol stress, WT plants exhibited more pronounced stress symptoms, including increased yellowing and wilting, compared to the transgenic lines (Figure 2A). Fresh weight analysis indicated that, while there was no significant difference under control conditions, under mannitol stress, the fresh weight of WT was significantly lower (0.38 ± 0.01 g) compared to the transgenic lines (around 0.67 g) (Figure 2B). Additionally, relative conductivity analysis showed higher values in WT (0.77 ± 0.02) compared to the transgenic lines (around 0.58) under mannitol stress (Figure 2C). These results demonstrate that *MsFtsH8* overexpression reduces water loss and cell membrane damage, further corroborating the enhanced drought resistance of *Arabidopsis* plants.

### 3.2. Drought Tolerance Analysis in MsFtsH8-Overexpressing Alfalfa

**Figure 3:**
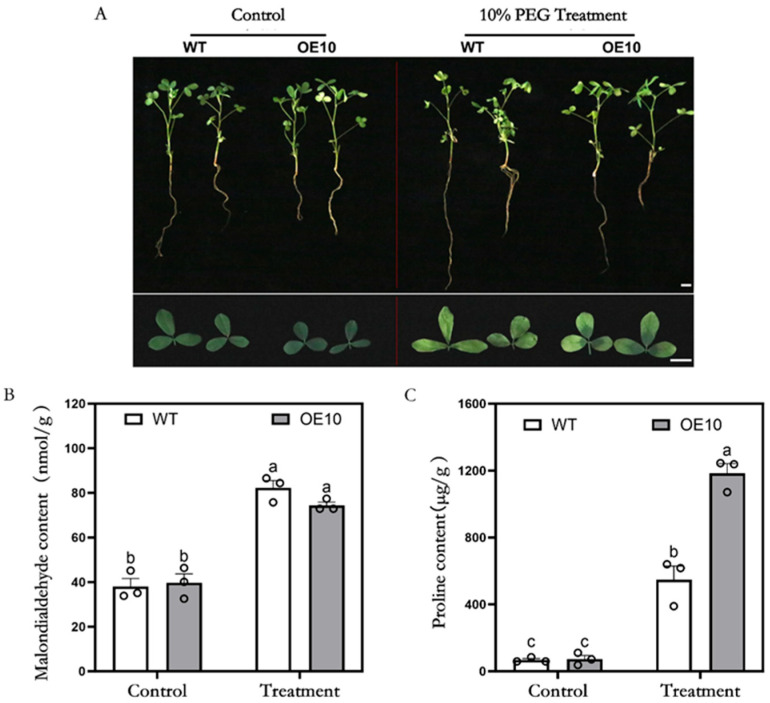
Growth phenotypes of alfalfa plants overexpressing *MsFtsH8* under PEG treatment. (**A**) Growth phenotype of alfalfa plants overexpressing *MsFtsH8* under PEG stress; (**B**) malondialdehyde content of alfalfa plants overexpressing *MsFtsH8* under PEG stress; (**C**) statistics of proline content of alfalfa overexpressing *MsFtsH8* under PEG Stress. Seventy-day-old alfalfa cuttings with uniform growth were treated with 1/2 Hoagland nutrient solution supplemented with 10% PEG 6000 for three days. The bar value in the lower right corner of (**A**) is 1 cm. The circles in this figure represent the value of each replicate; n = 3 for all groups. Bars marked with different lowercase letters denote statistically significant differences at *p* < 0.05 as assayed by ANOVA.

To assess the drought tolerance of *MsFtsH8*-overexpressing alfalfa, we subjected WT and OE10 plants to 10% PEG under hydroponic conditions. Both WT and OE10 plants exhibited normal growth under control conditions, but growth was restricted after PEG treatment, with leaves turning yellow, dehydrating, and wilting. However, OE10 plants displayed milder symptoms, with better growth and less water loss compared to WT (Figure 3A). Physiological analysis revealed that, while there was no significant difference in malondialdehyde (MDA) content under control conditions, PEG treatment significantly increased MDA levels in both genotypes. Notably, MDA content in WT (82.25 ± 3.31 nmol/g) was significantly higher than in OE10 (74.41 ± 1.49 nmol/g) (Figure 3B). Proline content analysis showed a significant increase in both genotypes under PEG stress, with OE10 (1148.35 ± 56.84 μg/g) exhibiting significantly higher levels than WT (548.77 ± 80.11 μg/g) (Figure 3C). These findings suggest that *MsFtsH8* overexpression mitigates lipid peroxidation and enhances osmotic stress regulation, thereby improving alfalfa’s drought tolerance.

### 3.3. Reactive Oxygen Species Levels in MsFtsH8-Overexpressing Alfalfa

**Figure 4:**
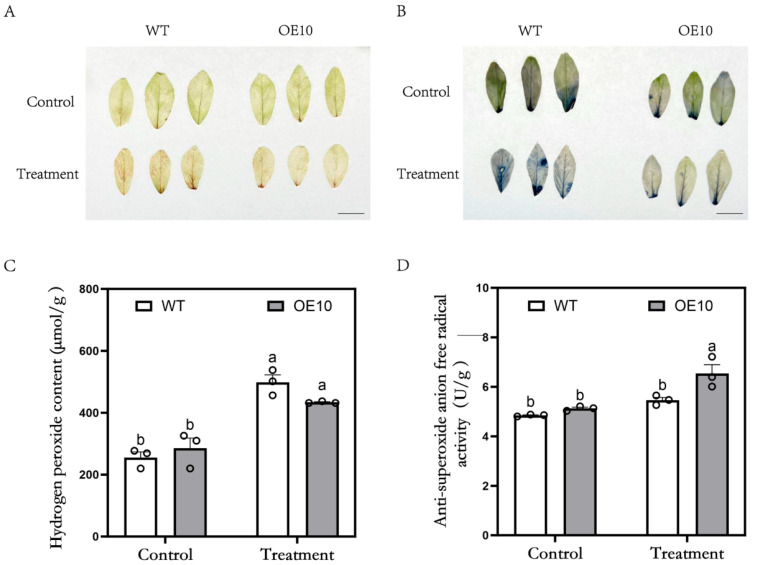
ROS activities in alfalfa plants overexpressing *MsFtsH8* under PEG treatment. (**A**) Dyed leaves with DAB. (**B**) Dyed leaves with NBT. (**C**) Hydrogen peroxide content of alfalfa plants overexpressing *MsFtsH8* under PEG stress. (**D**) Activity statistics of anti-superoxide anion free radical resistance of alfalfa overexpressing *MsFtsH8* under PEG stress. Seventy-day-old alfalfa cuttings with uniform growth were treated with 1/2 Hoagland nutrient solution supplemented with 10% PEG 6000 for three days. The bar value in the lower right corner of (**A**) is 1 cm. The circles in this figure represent the value of each replicate; n = 3 for all groups. Bars marked with different lowercase letters denote statistically significant differences at *p* < 0.05 as assayed by ANOVA.

To investigate the effect of *MsFtsH8* overexpression on ROS levels in alfalfa under drought stress, we conducted DAB and NBT staining, alongside measurements of the H_2_O_2_ content and anti-superoxide anion free radical activity. Under control conditions, no significant difference was observed in DAB and NBT staining between WT and OE10. However, following PEG stress, WT leaves exhibited darker staining compared to OE10 (Figure 4A,B), indicating higher ROS accumulation. H_2_O_2_ content analysis showed a significant increase in both genotypes after PEG treatment, with WT (498.61 ± 23.57 μmol/g) exhibiting higher levels than OE10 (433.68 ± 1.73 μmol/g) (Figure 4C). The analysis of anti-superoxide anion free radical activity indicated a remarkable increase in OE10 under PEG stress. Specifically, OE10 (6.54 ± 0.36 U/g) exhibited higher activity than WT (5.46 ± 0.11 U/g) (Figure 4D) after treatment. These results indicate that *MsFtsH8* overexpression brings down ROS accumulation and oxidative damage in alfalfa, thereby enhancing drought tolerance.

### 3.4. Antioxidant Enzyme Activities in MsFtsH8-Overexpressing Alfalfa

**Figure 5:**
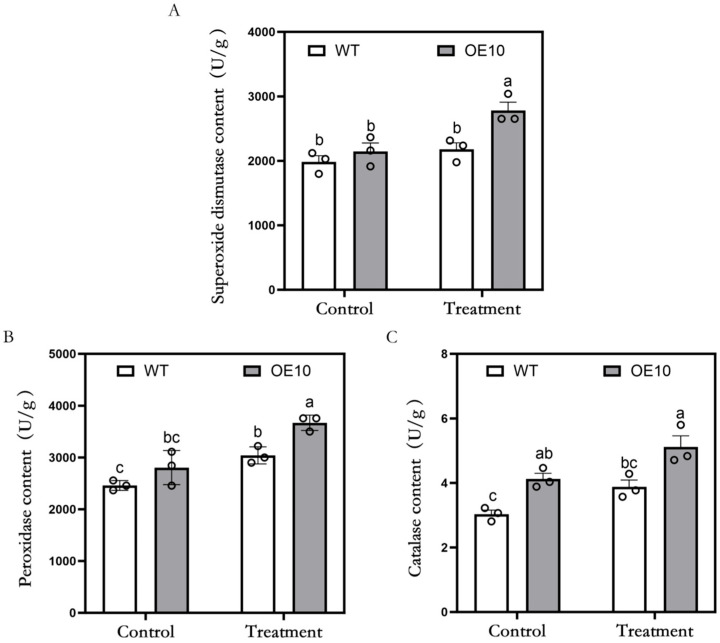
Activities of antioxidant enzymes in alfalfa plants overexpressing *MsFtsH8* under PEG stress. (**A**) Superoxide dismutase levels of alfalfa plants overexpressing *MsFtsH8* under PEG stress. (**B**) Statistics of peroxidase content of alfalfa plants overexpressing *MsFtsH8* under PEG stress. (**C**) Statistics of catalase content in alfalfa plants overexpressing *MsFtsH8* under PEG stress. Seventy-day-old alfalfa cuttings with uniform growth were treated with 1/2 Hoagland nutrient solution supplemented with 10% PEG 6000 for three days. The circles in this figure represent the value of each replicate; n = 3 for all groups. Bars represent SE. Bars marked with different lowercase letters denote statistically significant differences at *p* < 0.05 as assayed by ANOVA.

To determine whether *MsFtsH8* overexpression affects antioxidant enzyme activities in alfalfa under drought stress, we measured the activities of SOD, POD, and CAT. Under control conditions, SOD content was approximately 2065 U/g in both WT and OE10. Following PEG treatment, SOD content increased significantly in OE10 (2780.88 ± 129.34 U/g) (Figure 5A). POD content was approximately 2631.48 U/g in both genotypes under control conditions. However, after PEG treatment, POD content in OE10 increased to 3670.37 ± 85.19 U/g, significantly higher than WT (3040.74 ± 95.22 U/g) (Figure 5B). CAT content analysis showed that under control conditions, OE10 had significantly higher CAT levels (4.13 ± 0.17 U/g) compared to WT (3.04 ± 0.12 U/g). After PEG treatment, CAT content increased in both genotypes, with OE10 (5.12 ± 0.34 U/g) again showing significantly higher levels than WT (3.89 ± 0.21 U/g) (Figure 5C). These findings indicate that *MsFtsH8* overexpression significantly enhances antioxidant enzyme activities, reducing ROS levels and oxidative damage, thereby improving alfalfa’s drought resistance.

### 3.5. Expression Level Analysis of Genes Encoding Antioxidant Enzymes

**Figure 6:**
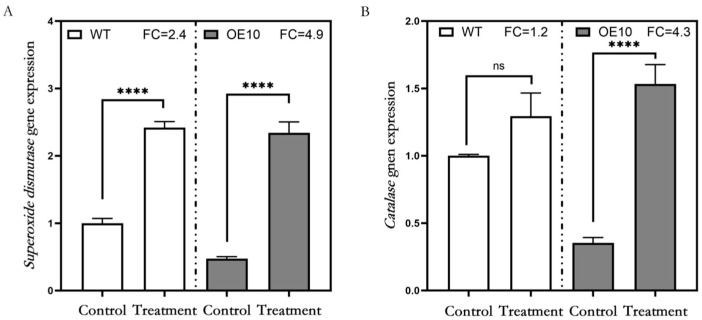
Analysis of gene expression of antioxidant enzymes in alfalfa overexpressing *MsFtsH8* under PEG stress. (**A**) *Superoxide dismutase* gene expression in alfalfa plants overexpressing *MsFtsH8* under PEG stress. (**B**) *Catalase* gene expression in alfalfa plants overexpressing *MsFtsH8* under PEG stress. Seventy-day-old alfalfa cuttings with uniform growth were treated with 1/2 Hoagland nutrient solution supplemented with 10% PEG 6000 for three days. FC represents fold change, and ns stands for non-significant, **** denotes *p* < 0.0001. The circles in this figure represent the value of each replicate; n = 3 for all groups. Bars represent SE.

To investigate the differences in the antioxidant capacity of alfalfa plants overexpressing the *MsFtsH8* gene under drought stress, we analyzed the expression changes in genes encoding antioxidant enzymes. The results demonstrated a significant increase in the expression levels of *SOD* in both WT and OE10 plants following PEG treatment, with 2.4-fold and 4.9-fold increases, respectively (Figure 6A). Similarly, the expression of *CAT* genes increased in both WT and OE10 plants after PEG treatment. However, while the increase in WT was only 1.2-fold and not statistically significant, OE10 exhibited a significant 4.3-fold increase (Figure 6B). These changes in gene expression correlated with the enzyme activity levels observed, confirming that the overexpression of the *MsFtsH8* gene enhances the antioxidant capacity of alfalfa, thereby improving drought stress.

## 4. Discussion

Drought stress is a crucial environmental factor that limits the growth and yield of alfalfa. Identifying key genes that confer drought tolerance is essential for genomic selection and genetic modification in alfalfa breeding programs. Previous research has identified *MsFtsH8* as a key gene that enhances salt stress tolerance by boosting the antioxidant system in *Arabidopsis* and alfalfa [1,17]. In this study, we assessed the role of *MsFtsH8* in conferring drought tolerance in *Arabidopsis* and alfalfa and explored the underlying mechanisms.

*FtsH* genes have been involved in multiple abiotic stress responses, such as salt, cold, and heat stress, across different plant species [1,12,13,19]. However, their role in drought tolerance has been less studied and is mainly reported in pepper (*Capsicum annuum* L.) [20]. Besides the proteolytically active FtsH proteases, pseudo-proteases, known as FtsHi (i for inactive [21]), have also been linked to drought tolerance, as seen in the *AtFTSHi3* knock-down mutant (*ftshi3*-*1(kd*)), which showed enhanced drought tolerance [22]. However, the functional role of *FtsH* genes in drought stress is largely unexplored.

Our recent work in *Medicago* demonstrated that the *FtsH* gene family is induced under various stress conditions, including the application of the stress-related phytohormone ABA [1,23]. Specific members such as *MtFtsH1*, *MtFtsH2*, *MtFtsH4*, *MtFtsH9*, and *MtFtsH10* in *Medicago truncatula*, and *MsFtsH3*, *MsFtsH4*, *MsFtsH9*, *MsFtsH13*, and *MsFtsH15* in alfalfa were upregulated in response to PEG treatment [1,23], suggesting their roles in drought stress defense. In this study, *MsFtsH8*-overexpressing plants exhibited better growth, higher fresh weight, improved membrane integrity, stronger osmotic adjustment, reduced cellular damage, lower ROS levels, and enhanced antioxidant capacity compared to WT plants—all of which are closely associated with drought tolerance [5,24]. This indicates the vital role of *MsFtsH8* in improving the drought tolerance of both *Arabidopsis* and alfalfa.

ROS, such as O_2_^−^, H_2_O_2_, OH·, and ^1^O_2_, play dual roles in plants, acting as both signaling molecules in growth and development and as mediators of oxidative stress under abiotic conditions [24]. Excessive ROS production under drought stress can lead to oxidative damage, affecting cell components and membranes [25,26]. The primary target of FtsH proteases, the D1 protein, is closely associated with oxidative stress resistance [27]. The loss of *FtsH* function can lead to elevated ROS levels due to the accumulation of damaged proteins and the disruption of protein complexes in chloroplasts and mitochondria [12,28,29], resulting in increased oxidative damage. In this study, the overexpression of *MsFtsH8* in alfalfa resulted in lower ROS levels, as evidenced by reduced H_2_O_2_ content and milder DAB and NBT staining following drought stress. This suggests that *MsFtsH8* is crucial for maintaining ROS balance in alfalfa under drought conditions.

Plants employ antioxidant enzymes like SOD, CAT, and POD to mitigate ROS toxicity and prevent oxidative damage [25,30]. The *MsFtsH8*-overexpressing alfalfa plants exhibited lower ROS levels, higher antioxidant enzyme activity, and an increased expression of genes encoding these enzymes under drought stress, consistent with their performance under salt stress [1]. These findings indicate that *MsFtsH8* enhances oxidative stress tolerance by boosting antioxidant enzyme activity, thereby reducing ROS levels during drought stress.

## 5. Conclusions

This study evaluated the growth and physiological performance of *MsFtsH8*-overexpressing plants under drought stress. The results revealed several advantageous traits in these plants, including better growth, improved membrane integrity, stronger osmotic adjustment, reduced cellular damage, lower ROS levels, and enhanced antioxidant capacity. These findings suggest that the overexpression of *MsFtsH8* improves drought tolerance in both *Arabidopsis* and alfalfa. The observed increases in the expression of key antioxidant enzyme-encoding genes further imply that the enhanced drought resistance in *MsFtsH8* overexpressing alfalfa is likely due to improved oxidative stress tolerance. This study confirms the role of *MsFtsH8* in conferring drought tolerance, primarily by boosting the ROS-scavenging capacity of antioxidant enzymes.

## Data Availability

Data are available within the article.
